# NEK4: prediction of available drug targets and common genetic linkages in bipolar disorder and major depressive disorder

**DOI:** 10.3389/fpsyt.2025.1414015

**Published:** 2025-01-30

**Authors:** Bin Gong, Chenxu Xiao, Yu Feng, Jing Shen

**Affiliations:** ^1^ The People’s Hospital of Danyang, Affiliated Danyang Hospital of Nantong University, Zhenjiang, China; ^2^ Department of Clinical Medicine, The Affiliated Jiangsu Shengze Hospital of Nanjing Medical University, Suzhou, China; ^3^ Department of Clinical Medicine, The University of Melbourne, Melbourne, VIC, Australia; ^4^ Department of Clinical Medicine, The University of New South Wales, Sydney, NSW, Australia

**Keywords:** major depressive disorder, genetic variants, drug targets, Mendelian randomization, *NEK4*

## Abstract

**Background:**

Bipolar disorder (BD) is a mental illness characterized by alternating episodes of elevated mood and depression, while major depressive disorder (MDD) is a debilitating condition that ranks second globally in terms of disease burden. Pharmacotherapy plays a crucial role in managing both BD and MDD. We investigated the genetic differences in populations of individuals with MDD and BD, and from a genetic perspective, we offered new insights into potential drug targets. This will provide clues to potential drug targets.

**Methods:**

This study employed genome-wide association studies (GWAS) and summary-data-based Mendelian randomization (SMR) methods to investigate the genetic underpinnings of patients with bipolar disorder (BD) and major depressive disorder (MDD) and to predict potential drug target genes. Genetic variants associated with BD and MDD were identified through large-scale GWAS datasets. For BD, the study utilized a comprehensive meta-analysis comprising 57 BD cohorts from Europe, North America, and Australia, including 41,917 BD cases and 371,549 controls of European ancestry. This dataset included both type 1 and type 2 BD cases diagnosed based on DSM-IV, ICD-9, or ICD-10 criteria through standardized assessments. For MDD, we used data from a meta-analysis by Howard DM et al., which integrated the largest GWAS studies of MDD, totaling 246,363 cases and 561,190 controls. The SMR approach, combined with expression quantitative trait loci (eQTL) data, was then applied to assess causal associations between these genetic variants and gene expression, aiming to identify genetic markers and potential drug targets associated with BD and MDD. Furthermore, two-sample Mendelian randomization (TSMR) analyses were performed to explore causal links between protein quantitative trait loci (pQTL) and these disorders.

**Results:**

The SMR analysis revealed 41 druggable genes associated with BD, of which five genes appeared in both brain tissue and blood eQTL datasets and were significantly associated with BD risk. Furthermore, 45 druggable genes were found to be associated with MDD by SMR analysis, of which three genes appeared simultaneously in both datasets and were significantly associated with MDD risk. *NEK4*, a common drug candidate gene for BD and MDD, was also significantly associated with a high risk of both diseases and may help differentiate between type 1 and type 2 BD. Specifically, *NEK4* showed a strong association with BD (β brain=0.126, P FDR=0.001; βblood=1.158, P FDR=0.003) and MDD (β brain=0.0316, P FDR=0.022; βblood=0.254, P FDR=0.045). Additionally, *NEK4* was notably linked to BD type 1 (βbrain=0.123, P FDR=2.97E-05; βblood=1.018, P FDR=0.002), but showed no significant association with BD type 2.Moreover, TSMR analysis identified four proteins (BMP1, F9, ITIH3, and SIGIRR) affecting the risk of BD, and PSMB4 affecting the risk of MDD.

**Conclusion:**

Our study identified *NEK4* as a key gene linked to both bipolar disorder (BD) and major depressive disorder (MDD), suggesting its potential as a drug target and a biomarker for differentiating BD subtypes. Using GWAS, SMR, and TSMR approaches, we revealed multiple druggable genes and protein associations with BD and MDD risk, providing new insights into the genetic basis of these disorders. These findings offer promising directions for precision medicine and novel therapeutic strategies in mental health treatment.

## Highlights

- Question: What are the possible potential drug targets for bipolar disorder and major depression?
- Findings: High expression of *NEK4* was associated with a high risk of bipolar disorder (β-brain = 0.126, β-blood = 1.158) and major depression (β-brain = 0.0316, β-blood = 0.254).
- Meaning: This implies that *NEK4* as a potential drug target may contribute to the treatment of bipolar disorder and major depression.

## 1 Introduction

Bipolar disorder (BD) is a mental illness characterized by alternating episodes of elevated mood and depression, negatively impacting the lives of a majority of those affected, despite its association with creativity (1). BD has the highest suicide rate among all mental disorders, approximately 20-30 times higher than that of the general population (2). However, the diagnosis of BD often presents challenges, as it can be easily confused with major depressive disorder (MDD). On a global scale, MDD affects over 350 million individuals and is a long-term disabling condition, ranking second in terms of disease burden worldwide (3). MDD is characterized by persistent and prolonged depressive states, accompanied by a loss of interest in daily activities, significantly impairing social and occupational functioning (4). It remains one of the most burdensome diseases globally.

Pharmacotherapy plays a pivotal role in managing both BD and MDD. For BD, common medications such as lithium, antidepressants, and antipsychotics are used to stabilize mood and prevent mood swings (5). However, due to individual variations and complex genetic backgrounds, patients exhibit substantial differences in their response to and tolerance of medication. While certain patients may respond well to a particular medication, others may experience treatment failure or severe side effects (6). Therefore, the development of precise and individualized drug therapy strategies is crucial to enhance treatment outcomes and improve the quality of life for patients.

Genomic association studies (GWAS) represent an advanced genetic approach that involves comprehensive analysis of genomic data from large populations (7), leading to the discovery of numerous genetic loci associated with BD and major depression (8, 9). These loci may be involved in various biological processes, including neurotransmitter pathways, neurodevelopment, and immune response (10) However, GWAS studies often face challenges in establishing causal relationships between these genetic variants and specific genes, genomic regions, and disease phenotypes.

Mendelian randomization (MR) is a statistical method that utilizes genetic variation to assess causality. It capitalizes on the random and independent effect of genetic variation on individuals, simulating clinical trials, and thereby determining whether a causal relationship exists between a factor and a disease (11). Summary data-based Mendelian randomization (SMR) extends this approach by incorporating summary data, combining GWAS and expression quantitative trait loci (eQTL) data to evaluate causal associations between gene expression and disease (12). The application of SMR allows us to pinpoint genetic markers associated with BD and MDD more accurately and predict potential drug targets, offering new avenues and strategies for personalized treatment.

This study aimed to delve into the genetic underpinnings of BD and MDD patients using GWAS, SMR, and Two-Sample Mendelian Randomization (TSMR) methods. By identifying relevant genetic factors and evaluating their causal roles, we seek to deepen the biological understanding of these disorders, which may guide future research on personalized therapeutic approaches and drug target development.

## 2 Method

### 2.1 Identification of druggable genes

Druggable genes were identified using the Drug-Gene Interaction Database (DGIdb V.4.2.0, https://www.dgidb.org/), a comprehensive resource that provides information on drug-gene interactions and druggable genes through publications, databases, and web-based sources (13). Specifically, we downloaded the “category data” from DGIdb (released in February 2022), which includes all genes categorized as druggable and mapped to Entrez genes (14).

### 2.2 BD and MDD GWAS data

The study utilized the most extensive BD GWAS dataset, comprising a meta-analysis of 57 BD cohorts from Europe, North America, and Australia. This dataset encompassed a total of 41,917 BD cases and 371,549 controls of European ancestry, including combined data from type 1 and type 2 BD cases. Cases in this dataset fulfilled the international consensus criteria for lifelong BD (DSM-IV, ICD-9, or ICD-10), which were established through structured diagnostic interviews, clinician-administered checklists, or chart reviews (15). As for the MDD GWAS data, it was derived from a meta-analysis conducted by Howard DM et al., which involved the amalgamation of data from the three largest genome-wide association studies of MDD. The MDD GWAS dataset included a total of 807,553 individuals (246,363 MDD cases and 561,190 controls) (16).

### 2.3 Summary data-based Mendelian randomization analysis

The SMR software tool was originally developed to implement the SMR & HEIDI method, which effectively combines data from GWAS and expression quantitative trait loci (eQTL) studies to assess pleiotropic associations between gene expression levels and complex traits of interest (12). For LD calculations, we utilized the 1000 Genomes European reference data (17).

Regarding brain tissue eQTL data, we conducted eQTL mapping by employing RNA-seq data obtained from 2,865 cerebral cortex samples from 2,443 unrelated individuals of European ancestry, along with genome-wide SNP data, leading to the identification of cis-eQTL information specific to brain tissue (18). As for blood eQTL data, it was derived from the eQTLGen consortium, encompassing information on 10,317 single nucleotide polymorphisms (SNPs) related to traits from a cohort of 31,684 individuals (19). To validate our findings, we cross-referenced with whole blood cis-eQTL data from the Genotype-Tissue Expression Project (GTEx) V.8, accessible through the website (https://www.gtexportal.org), which provides details on donor registration, consent procedures, biospecimen procurement, sample fixation, and other pertinent information (20). The false discovery rate (FDR) for the P-value of SMR was calculated using the BH method, and we set FDR SMR < 0.05 and heterogeneity HEIDI > 0.01 as criteria for inclusion in the results.

### 2.4 Two-sample Mendelian randomization analysis

To delve deeper into the association between protein quantitative trait loci (pQTL) and these two psychiatric disorders, we employed the Two-Sample Mendelian Randomization (TSMR) method for analysis. TSMR analysis was performed using the “TwoSampleMR” package in Rstudio (version 4.3.0). The main analysis method utilized for causal assessment between pQTL and the two psychiatric disorders was Inverse Variance Weighted (IVW) (21).

In this analysis, pQTL-associated SNP proposals were used as instrumental variables, and we identified SNPs significantly associated with pQTL by setting the genome-wide significance threshold at p < 5 × 10^-8. To account for the potential influence of linkage disequilibrium (LD) on the results, we required that SNPs significantly associated with an exposure should have an LD with r^2 < 0.01 and a KB > 5000. We applied Bonferroni corrections to address multiple testing, and the results were filtered using a threshold p-value of 0.05/1519 (p < 3.29 × 10^-5).

The data on cerebrospinal fluid pQTL originated from a study conducted by Yang et al., which reported 274 pQTL associated with 184 CSF proteins (22). Plasma pQTL data were obtained from a study by Cheng et al., encompassing 738 cis-acting SNPs related to 734 proteins (23). Additionally, plasma pQTL data from Ferkingstad et al. were also included in this study, encompassing measurements of 4907 plasma proteins in 35559 Icelandic participants (24).

### 2.5 Brain function and structure associated with BD and MDD

**Figure 1:**
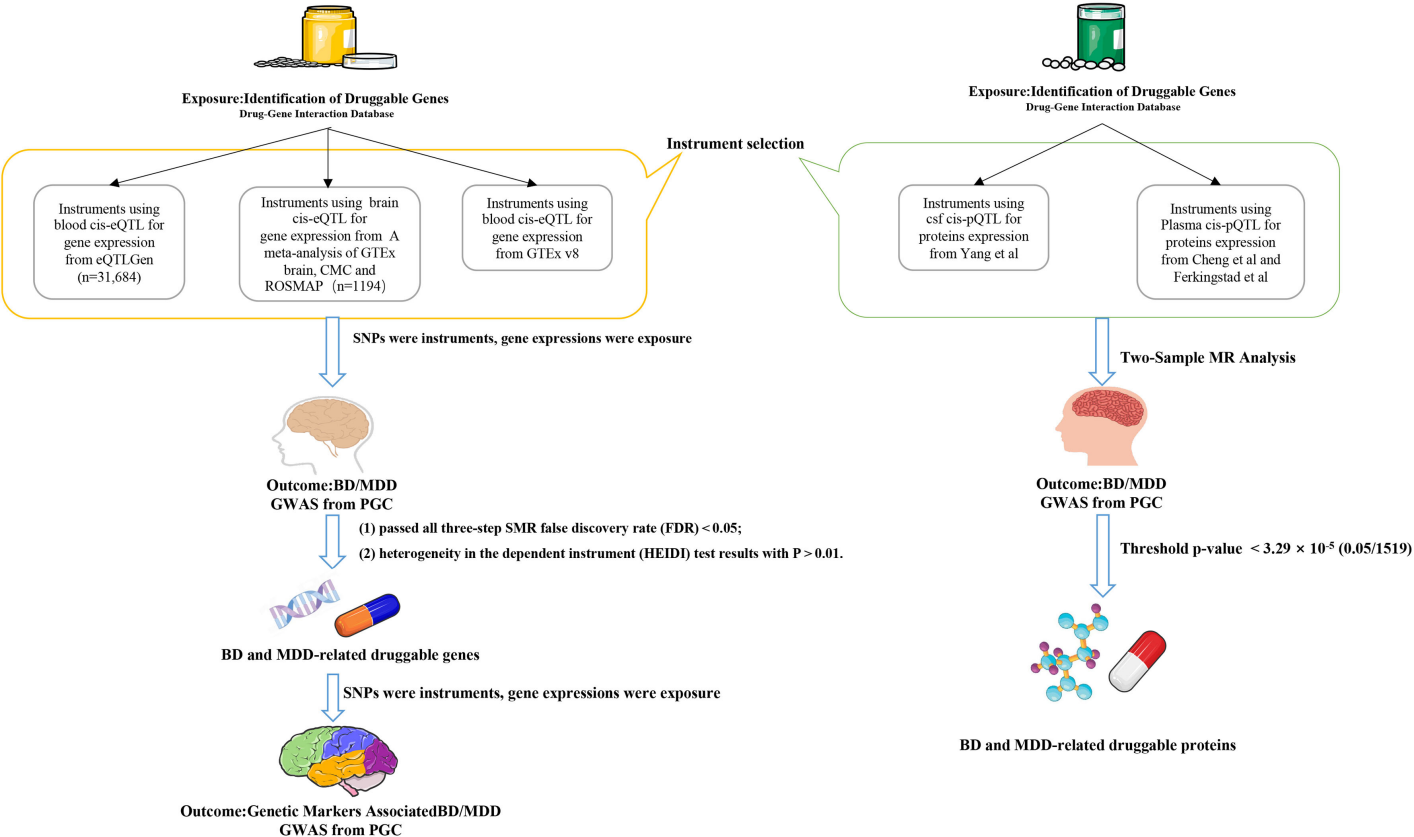
Flow chart. The flowchart illustrates the analytical process of this study. The left pathway represents the SMR analysis of the relationship between eQTLs and MDD/BD, while the right pathway represents the TSMR analysis of the relationship between pQTLs and MDD/BD. TSMR, Two-Sample Mendelian Randomization Analysis; SMR, Summary Data-Based Mendelian Randomization () Analysis; BD, Bipolar Disorder; MDD, Major Depressive Disorder.

To validate whether disease-associated genes are related to brain structure and function, we performed SMR analysis using the same brain and blood eQTL datasets on brain function and structure (25, 26), We used brain function and structure GWAS as the exposure and BD/MDD GWAS as the outcome to explore the mediation effects. The brain function and structure GWAS data were sourced from Elleke et al.’s genetic architecture of functional and structural connectivity properties within the resting-state brain networks, encompassing seven networks (Dorsal Attention, Frontoparietal, Global, Limbic, Somatomotor, Ventral Attention, and Visual) for both structural (SC) and functional (FC) connectivity (27) (**Figure 1**).

### 2.6 Enrichment analysis

For the gene set functional enrichment analysis, we first downloaded the c5.go.cc.v7.4.symbols.gmt subset from the Molecular Signatures Database (http://www.gsea-msigdb.org/gsea/downloads.jsp) as the background (28). We then mapped the genes to the background set and performed enrichment analysis using the R package clusterProfiler (version 3.14.3) to obtain the gene set enrichment results. The minimum gene set size was set to 5, and the maximum gene set size to 5000. A P value of < 0.05 and a false discovery rate (FDR) of < 0.1 were considered statistically significant.

### 2.7 Ethical approval

The data used in this study were anonymized and de-identified and did not involve any direct contact or intervention with human or animal subjects. Therefore, no additional ethical approval was required for this study.

## 3 Results

### 3.1 BD drug candidate genes

A total of 3953 genes were identified as potential drug targets from the DGIdb. Among these, we queried the brain tissue eQTL data for 2239 genes and the blood eQTL data for 2869 genes. After validation using the GTEx blood eQTL data, 1554 druggable gene eQTL data were obtained.

**Figure 2:**
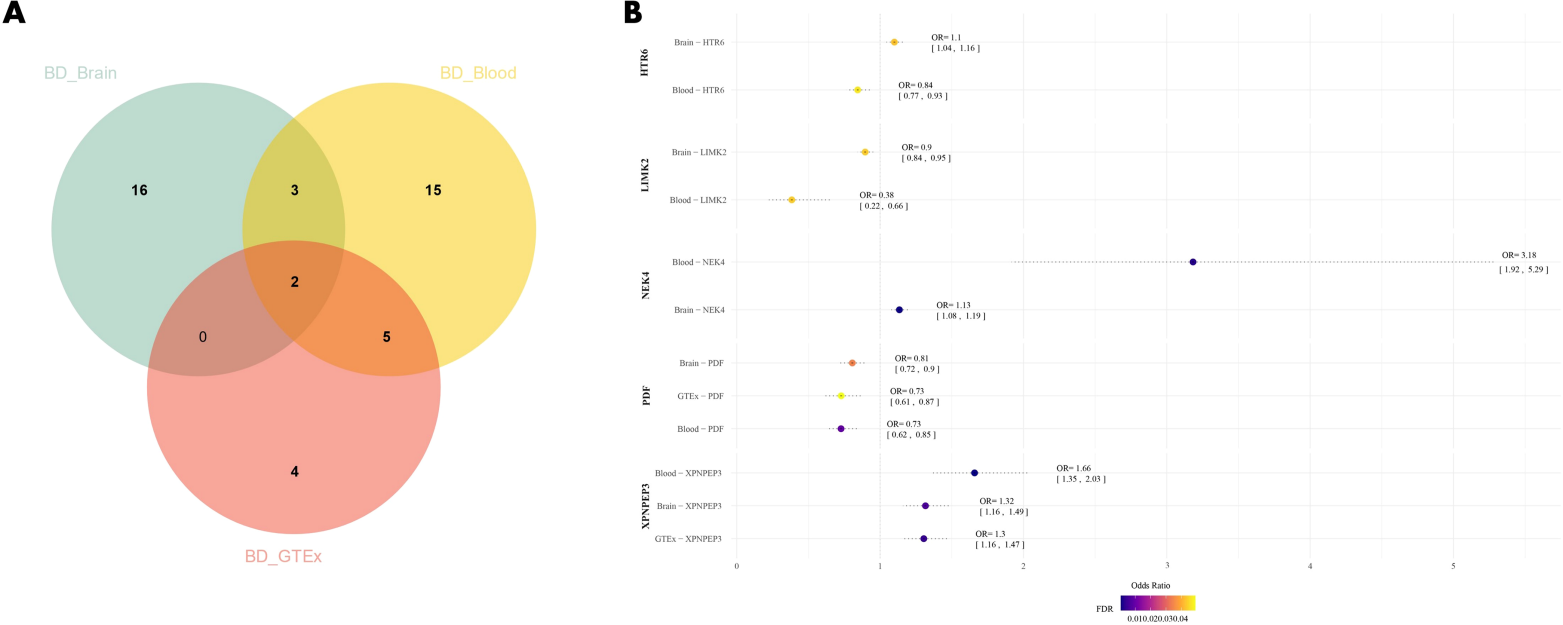
**(A)** Venn diagram of the intersection of three datasets; **(B)** Forest plot of BD drug candidate genes, dots are color coded according to significance level (-log10(FDR)), significant results are shown in darker colors. BD, Bipolar Disorder.

Through SMR analysis with BD, we found 41 druggable genes associated with BD, with 21 genes identified in brain tissue and 25 genes in blood (**Supplementary Tables 1**, **2**). Among these genes, five were present in both brain tissue and blood eQTL datasets (*HTR6, NEK4, PDF, LIMK2, XPNPEP3*). Furthermore, in the SMR analysis of GTEx blood eQTL with BD (**Supplementary Table 3**), two genes (*PDF* and *XPNPEP3*) were consistently found in all three datasets and were significantly correlated with the risk of BD (FDR<0.05). High expression of *PDF* may potentially reduce the risk of BD (βbrain=-0.215, βblood=-0.318, βGETx=-0.317), while high expression of *XPNPEP3* may potentially increase the risk of BD (βbrain=0.275, βblood=0.506, βGETx=0.266) (**Figure 2**).

### 3.2 MDD drug candidate genes

**Figure 3:**
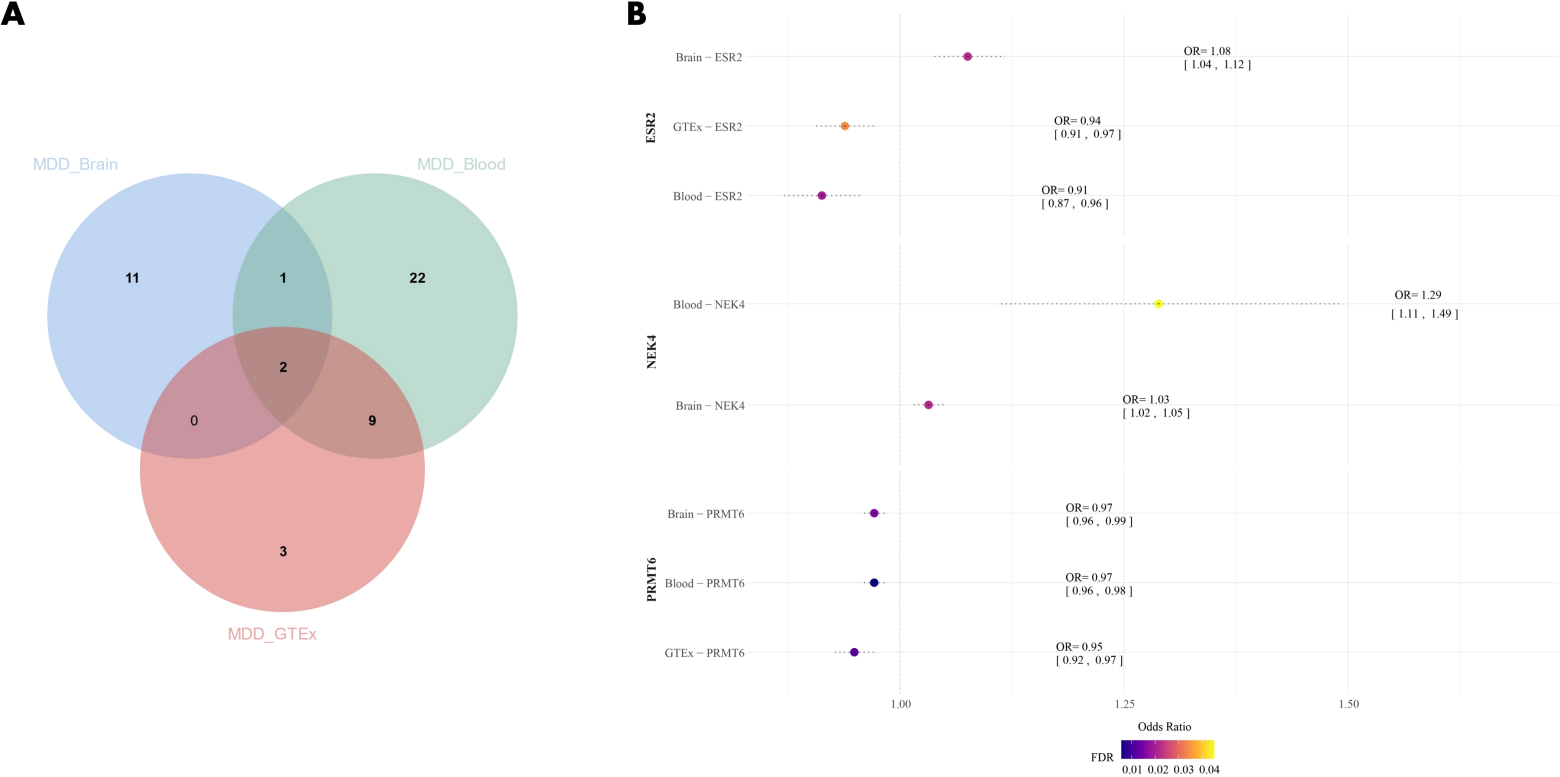
**(A)** Venn diagram of the intersection of three datasets; **(B)** Forest plot of MDD drug candidate genes, dots are color coded according to significance level (-log10(FDR)), significant results are shown in darker colors. MDD, Major Depressive Disorder.

Through SMR analysis of brain tissue and blood eQTL data, we identified 45 druggable genes associated with MDD, with 14 of them found in brain tissue and 34 in blood (**Supplementary Tables 4**, **5**). Among these, three genes (*PRMT6*, *NEK4*, and *ESR2*) were concurrently present in both datasets. In the validation group, both PRMT6 and *ESR2* showed a significant correlation (FDR<0.05) with MDD (**Supplementary Table 6**). However, no correlation was found between *NEK4* and MDD, likely due to the absence of eQTL data for *NEK4* in the GTEx dataset (**Figure 3**). High expression of *PRMT6* may potentially reduce the risk of MDD (βbrain=-0.0289, βblood=-0.0290, βGTEx=-0.0520). Notably, *ESR2* exhibited a positive correlation with MDD, except in brain tissue (β=0.0731), while it showed a negative correlation in both the other datasets (βblood=-0.0909, βGTEx=-0.0632).

### 3.3 BD and MDD common drug candidate genes

**Figure 4:**
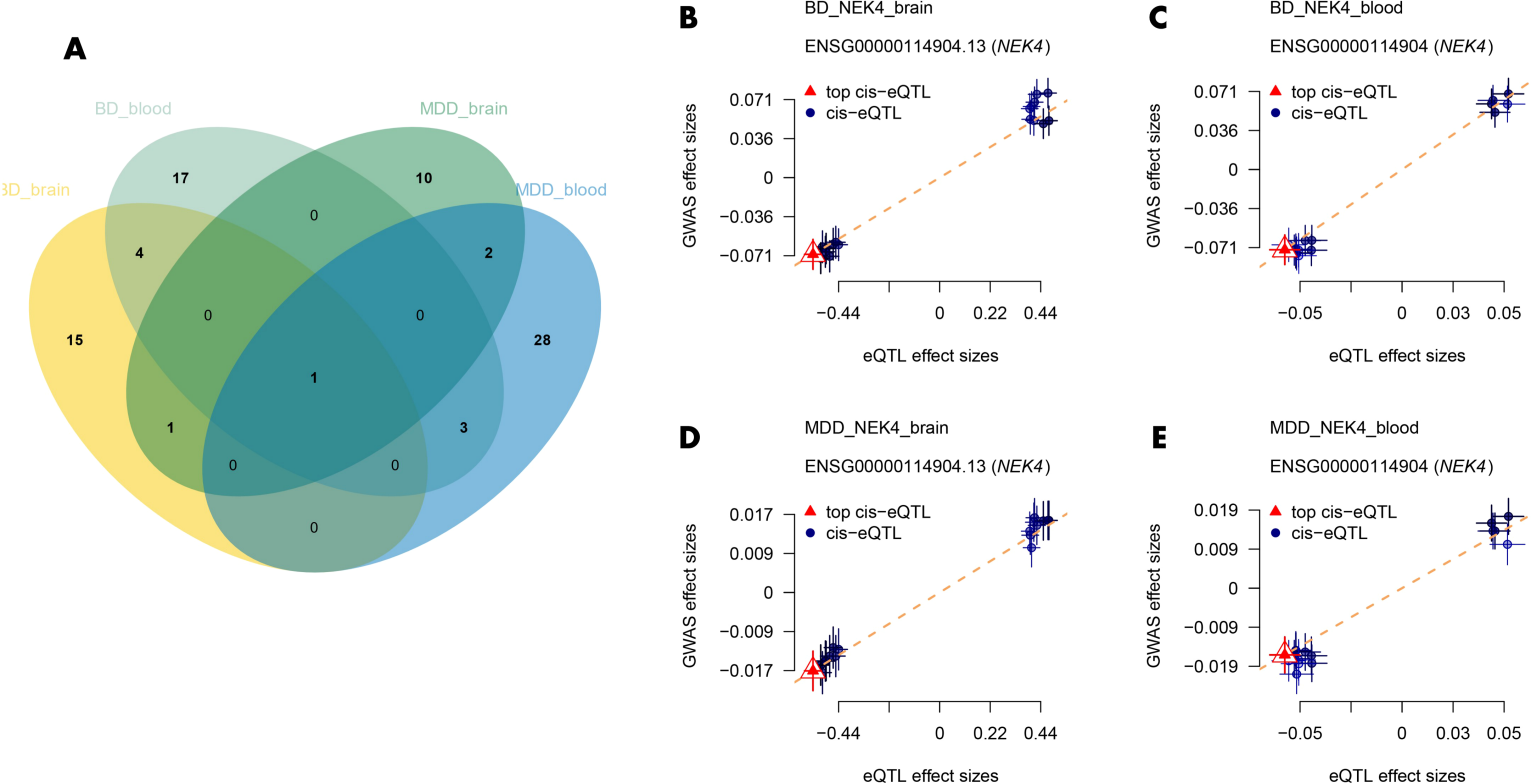
**(A)** Venn diagrams of common drug candidate genes in both datasets for BD and MDD; **(B–E)** Depiction of the causal relationship between NEK4 and BD/MDD. BD, Bipolar Disorder; MDD, Major Depressive Disorder.

In this step, we focused on identifying common drug candidate genes associated with both BD and MDD. Since the blood eQTL dataset of GTEx contains only half of the druggable genes, we excluded its results in this analysis. Instead, we examined the brain tissue dataset and found 2 genes associated with both BD and MDD (*NEK4* and *SLC12A5*). In the blood dataset, we identified 4 genes associated with both BD and MDD (*GNL3, NEK4, ITIH3*, and *DCBLD1*). Among these genes, *NEK4* displayed a correlation with both BD and MDD in both datasets (**Figure 4A**). High risk of both BD (βbrain=0.126, βblood=1.158) and MDD (βbrain=0.0316, βblood=0.254) was associated with high expression of *NEK4* (**Figures 4B–E**).

Additionally, we performed SMR analysis to explore the association of *NEK4* with different types of BD. The results revealed that high expression of *NEK4* was significantly associated with BD type 1 (βbrain=0.123, βblood=1.018) (**Supplementary Tables 7**, **8**), while no significant association was found with BD type 2 (FDR>0.05). This indicates that *NEK4* may serve as a potential genetic marker to differentiate between BD type 1 and type 2.

### 3.4 The association between BD/MDD drug genes and brain functional structure

In brain tissue, we did not find any significant association between two genes (*NEK4* and *SLC12A5*) and brain functional structure (**Supplementary Table 9**). However, in blood tissue, ITIH3 showed a potential correlation with the structure of the Dorsal Attention network, and DCBLD1 showed a potential correlation with the structure of the Frontoparietal network (p<0.05) (**Supplementary Table 10**). Moreover, when using markers as the exposure and BD/MDD as the outcome, both BD and MDD were found to have a potential correlation with the structure of the Dorsal Attention network (p<0.05), while no significant correlation was observed with the structure of the Frontoparietal network (**Table 1**).

**Table 1:** TSMR analysis of the association between BD/MDD and markers.

outcome	exposure	method	nsnp	or	or_lci95	or_uci95	pval	Q_pval
SC_Dorsal.Attention	BD	IVW	8	1.21E-18	2.21E-34	6.62E-03	2.57E-02	7.12E-02
SC_Dorsal.Attention	MDD	IVW	7	3.44E+07	9.18E+00	1.29E+14	2.46E-02	1.00E-02
SC_Frontoparietal	BD	IVW	12	1.50E-04	2.21E-20	1.03E+12	6.36E-01	5.53E-01
SC_Frontoparietal	MDD	IVW	10	5.86E-01	2.72E-10	1.27E+09	9.61E-01	1.24E-03

TSMR, Two-Sample Mendelian Randomization Analysis; BD, Bipolar Disorder; MDD, Major Depressive Disorder; IVW, Inverse Variance Weighted.

### 3.5 BD and MDD drug candidate proteins

For the cerebrospinal fluid, we examined 15 druggable pQTL data, and for plasma, we analyzed 1054 druggable pQTL data. By conducting TSMR analysis, we identified four proteins (BMP1, F9, ITIH3, and SIGIRR) that significantly affected BD risk, and PSMB4 that affected MDD risk. Specifically, high expression of BMP1 (OR=2.47, 95% CI, 1.69-3.62) and F9 (OR=2.02, 95% CI, 1.50-2.72) were found to increase the risk of BD, while high expression of ITIH3 (OR=0.84, 95% CI, 0.78-0.90) and SIGIRR (OR=0.91, 95% CI, 0.88-0.95) were associated with a decreased risk of BD. Additionally, high expression of PSMB4 (OR=1.04, 95% CI, 1.02-1.06) was identified to increase the risk of MDD (**Table 2**).

**Table 2:** TSMR analysis of drug candidate proteins for BD and MDD.

Group	Tissue	Protein	OR (95% CI)	P value	F statistics	Author
BD	Plasma	BMP1	2.47 (1.69, 3.62)	3.14E-06	22165139	Suhre
	Plasma	F9	2.02 (1.50, 2.72)	3.14E-06	139530719	Suhre
	Plasma	ITIH3	0.84 (0.78, 0.90)	5.02E-07	52794767	Suhre
	Plasma	SIGIRR	0.91 (0.88, 0.95)	4.15E-07	417454	Suhre
MDD	Plasma	PSMB4	1.04 (1.02, 1.06)	1.38E-05	151399564	Suhre

TSMR,Two-Sample Mendelian Randomization Analysis; BD, Bipolar Disorder; MDD, Major Depressive Disorder.

### 3.6 Enrichment analysis

Previous findings indicate a common association of genes such as *GNL3, NEK4, ITIH3, DCBLD1*, and *SLC12A5* with both MDD and BD. GO enrichment analyses for molecular function (MF), biological process (BP), and cellular component (CC) further highlighted several key functional terms linked to these genes. In the Molecular Function (MF) category, genes were significantly enriched in potassium chloride symporter activity, cation chloride symporter activity, anion-cation symporter activity, and mRNA 5’ untranslated region binding(**Supplementary Table 11**). In the Biological Process (BP) category, the enriched terms included cellular monovalent inorganic anion homeostasis, response to hypotonic stress, regulation of protein sumoylation, and cellular anion homeostasis. Notably, cellular processes such as cell division, protein localization, and potassium ion homeostasis were also highlighted(**Supplementary Table 12**). In the Cellular Component (CC) category, significant enrichment was observed in the ciliary rootlet, platelet dense granule lumen, and platelet dense granule. These findings indicate that the identified genes are involved in diverse molecular functions, biological processes, and cellular components, suggesting their potential roles in various physiological and cellular mechanisms (**Supplementary Table 13**).

## 4 Discussion

This study aims to delve deeper into the genetic foundations of BD and MDD patients using genome-wide association studies (GWAS), summary-data-based Mendelian randomization (SMR), and two-sample Mendelian randomization (TSMR) methods. It also seeks to make predictions about potential drug target genes, with the goal of providing more individualized and effective strategies for clinical treatment.

Initially, we identified druggable genes associated with BD and MDD from the DGIdb. Subsequently, we employed SMR analysis of brain tissue and blood eQTL data, using the largest BD and MDD GWAS dataset to validate the links between these druggable genes and the respective disorders. This process yielded a list of druggable genes associated with BD and MDD, including candidate genes found in both brain tissue and blood.

In the TSMR analysis, we delved further into the pQTL associated with these psychiatric disorders, uncovering several proteins linked to the risk of BD and MDD. The presence of these proteins suggests their potential involvement in the pathogenesis of psychiatric disorders. Specifically, high expression of BMP1 and F9 proteins were associated with an increased risk of BD, while high expression of ITIH3 and SIGIRR proteins were associated with a decreased risk of BD. Additionally, high expression of PSMB4 protein was found to be potentially associated with an increased risk of MDD. These findings offer valuable insights for the identification of future therapeutic targets.

Regarding the association markers, we discovered that both BD and MDD were associated with Dorsal Attention in brain structures, while the correlation with Frontoparietal was not significant. These results provide additional clues to unravel the relationship between psychiatric disorders and brain function and structure.

Furthermore, we identified the *NEK4* gene as being associated with both BD and MDD, and its high-risk alleles may be implicated in the pathogenesis of these two disorders. This suggests that individuals carrying specific *NEK4* genotypes may be more susceptible to developing BD or MDD compared to those with other genotypes. *NEK4* encodes a protein linked to cell cycle regulation and cell division (29), and it has been confirmed as a risk gene for BD by several eQTL analyses of various brain regions (30–33). However, the exact mechanism underlying the relationship between *NEK4* and BD and MDD remains unclear. The encoded protein may be involved in various neurobiological processes such as neurotransmitter regulation, neuronal function, and connectivity through multiple pathways, potentially contributing to BD and MDD pathogenesis (34, 35). The association between this gene and markers is not straightforward, indicating a complex pathway of its influence on mental diseases, warranting further in-depth research.

To better understand the biological pathways associated with BD and MDD, we performed Gene Ontology (GO) enrichment analysis on the identified genes. The enrichment results revealed associations with cellular components such as “ciliary root” and “platelet dense granule lumen,” as well as significant roles in neurotransmitter systems and signaling pathways. Notably, *NEK4* is involved in processes related to ciliary structure and function, which are linked to neuronal signal transduction and psychiatric disorders (36). *NEK4* is also closely associated with the regulation of cell structure and function. For instance, it plays a role in the stability of primary cilia and affects mitochondrial morphology and respiratory function (37). Furthermore, several GWAS have identified *NEK4* as a common risk gene for BD and schizophrenia (32). These findings provide insights into how *NEK4* may influence the potential cellular environment and biological processes in BD and MDD, particularly in cell cycle regulation and neural circuits.

Functional enrichment analysis highlighted pathways and cellular components that may be involved in neurodevelopmental and neuroinflammatory processes underlying BD and MDD, such as “ciliary root” and “platelet dense granules.” *NEK4*’s involvement in these pathways supports our hypothesis that it may affect disease risk through pathways involving neurotransmitter regulation and neuronal connectivity. The shared pathways between *NEK4* and other related genes emphasize the relevance of cellular structure and signaling in the pathogenesis of BD and MDD, suggesting that these pathways could serve as promising targets for future therapeutic strategies.

Overall, this study systematically investigated the genetic basis of BD and MDD patients using GWAS, SMR, and TSMR methods, and predicted potential drug target genes. By identifying drug genes and proteins associated with BD and MDD, we offer new potential strategies and drug targets for precision medicine in treating these two psychiatric disorders. However, it is important to note that the study has certain limitations, including sample size and data sources. In the analysis of tissue specificity, due to limitations in sample size and data, although some potential associations were found, these results still need to be further validated through larger samples or more refined analyses. Expanding the sample size and integrating more data sources would be necessary to validate the findings. Nevertheless, our study provides valuable insights and strategies for a comprehensive understanding of the genetic basis of BD and MDD, and for future individualized therapies.

## 5 Conclusion

Our study provides a comprehensive genetic analysis of BD and MDD using GWAS, SMR, and TSMR methodologies to identify potential drug target genes. Significantly, *NEK4* emerged as a common gene associated with increased risk in both BD and MDD, highlighting its potential as a drug target and as a biomarker that may help differentiate between BD types 1 and 2. *NEK4* demonstrated a notable association with BD risk, particularly for BD type 1, as well as a consistent association with MDD. The identification of multiple druggable genes and causal links between proteins and these psychiatric conditions enhances our understanding of the molecular pathways underlying BD and MDD. Our findings underscore the importance of *NEK4* and other identified genes as potential targets for precision medicine, offering a new direction for therapeutic strategies that could improve patient outcomes and advance mental health treatment.

## Data Availability

The original contributions presented in the study are included in the article/**Supplementary Material**. Further inquiries can be directed to the corresponding author.
